# Forced Expression of Foxg1 in the Cortical Hem Leads to the Transformation of Cajal-Retzius Cells into Dentate Granule Neurons

**DOI:** 10.3390/jdb6030016

**Published:** 2018-06-26

**Authors:** Bin Liu, Hongmei Xiao, Chunjie Zhao

**Affiliations:** Key Laboratory of Developmental Genes and Human Diseases, MOE, School of Medicine, Southeast University, Nanjing 210009, China; myqj2006@126.com (B.L.); xhmforever@hotmail.com (H.X.)

**Keywords:** cortical hem, Cajal-Retzius cell, cortical patterning, Foxg1, Lhx2, hippocampus, granule cell, Wnt

## Abstract

The Wnt- and BMP-rich cortical hem has been demonstrated to be critical for the pattern formation of the telencephalon, and it is particularly important for the induction of the hippocampus. Meanwhile, the cortical hem is one of the sources of Cajal-Retzius cells. Many Cajal-Retzius cells are produced in the hem and populated to the media-caudal surface of the telencephalon. However, the mechanism of the maintenance of the hem remain unclear. In this study, we generated a transgenic mouse line *CAG-loxp-stop-loxp-Foxg1-IRES-EGFP*. By crossing *Fzd10CreER^TM^* with this line, combined with tamoxifen induction, Foxg1 was ectopically expressed in the hem from embryonic day 10.5 (E10.5) onwards. We have found the hem-derived Cajal-Retzius cells were transformed into dentate granule neurons accompanied with ectopic expression of Lhx2. However, the morphology of the hem displayed no obvious changes. The hem specific markers, Wnt3a and Wnt2b, were slightly downregulated. Our results indicate that Foxg1 is sufficient to induce the expression of Lhx2 in the dorsal part of the hem. The ectopic Lhx2 and decreased Wnt signals may both contribute to the cell fate switch. Our study provides new insight into the mechanism underlying the maintenance of the hem.

## 1. Introduction

During the cortical development, regionalization of the cortex is controlled by morphogens secreted from signal centres located at the perimeter of the telencephalon and transcription factors expressed in a gradient pattern along the coordinate axes [1,2]. Regionalization along the medio-lateral axis leads to the progressive subdivision of the telencephalic neuroepithelium into the medial pallium, which gives rise to the hippocampus, including the dentate gyrus (DG), the adjacent dorsal pallium that gives rise to the neocortex, respectively. The Wnt- and BMP-rich cortical hem, located between the telencephalic choroid plexus and hippocampal primordium, has been identified to be one of the cortical organizers to regulate the patterning of the telencephalon [3,4]. A severely-reduced neocortex, particularly dorsomedial neocortex was reported after ablation of the hem [5]. Disruption of *Wnt3a*, a gene specifically expressed in the hem, leads to loss of the whole hippocampus [6]. When *Lef1*, a downstream mediator of Wnt signalling, is deleted, the development of the hippocampus is significantly affected [7]. Previous study also shows that an ectopic hem is capable to induce an ectopic hippocampus [8]. Thus, the cortical hem is necessary and sufficient to induce the adjacent hippocampal primordium. Meanwhile, cell linage mapping has revealed the hem is one of the sources of Cajal-Retzius (CR) cells, the cell population which is crucial for the cortical lamination [9,10,11,12]. Despite its critical roles, the mechanism of regulating the hem during the telencephalic development is still unclear.

The transcription factor *Foxg1*, formerly called Brain Factor1, is strongly expressed in the telencephalon, but specifically excluded from the hem. Its expression can be detected as early as E8.5 [13]. Previously, *Foxg1* and the transcription factor *Lhx2* have been shown to play important roles to suppress the hem. Both *Foxg1* and *Lhx2* null mutants exhibit significant expansion of the hem [14,15]. Recently, Godbole and colleagues showed that *Foxg1* functions upstream of *Lhx2* to regulate the specification and positioning of the hem, providing new insight into the mechanisms regulating the hem formation [16]. On the other hand, there are obvious phenotype differences between *Foxg1* and *Lhx2* knockouts. The hem expands to the whole cortex accompanied with a complete loss of the dorsal pallium after constitutive disruption of *Lhx2* [8], while part of the hippocampal primordium remains in the *Foxg1*-deficient telencephalon [13,17], suggesting a complicated mechanism of *Foxg1* and *Lhx2* to regulate the hem. Meanwhile, the hem itself displays a heterogeneous feature with its dorsal and ventral parts displaying distinct expression patterns of Wnts and BMPs [4], and the more detailed mechanism underlying the maintaining of the hem still needs to be further elucidated. 

The cortical hem forms before cortical neurogenesis [3,4]. Although constitutive loss of Foxg1 from the very early developmental stage results in a severe expansion of the hem, removal of Foxg1 at E13 does not cause the hem expansion [18], suggesting Foxg1 may regulate the hem in a spatiotemporal manner. In this study, we focused on the role of Foxg1 during the time window of E10.5 onwards when the formation of the hem is almost completed. We have generated a transgenic mouse line *CAG-loxp-stop-loxp-Foxg1-IRES-EGFP* in which *Foxg1* cDNA is driven by *CAG* promoter, and the overexpression commenced upon the deletion of stop codon by Cre medicated recombination. By crossing with *Fzd10CreER^TM^*, Foxg1 was ectopically overexpressed in the hem. We found a large population of the hem-derived CR cells switched their fate into dentate granule neurons, accompanied with upregulation of Lhx2, suggesting Foxg1 is sufficient to induce Lhx2 and specify granule cell fates. However, it seems the morphology of the hem was normal with only slightly decreased expression levels of *Wnt3a* and *Wnt2b*. Our study will help to understand the mechanism underlying the maintenance of the hem.

## 2. Materials and Methods

### 2.1. Generation of *CAG-loxp-stop-loxp-Foxg1-IRES-EGFP* Mouse Line

*Foxg1* cDNA was subcloned into an *IRES2-EGFP* vector (Addgene, cat6029-1, Cambridge, MA, USA). The *loxp-stop-loxp* fragment was inserted ahead of the *Foxg1* cDNA fragment. *CAG* promoter was cut out from a *pCAGIG* vector and inserted into the *loxp-stop-loxp-Foxg1-IRES-EGFP* vector. The transgenic construct was then linearized, purified with a gel extraction kit (Qiagen, 20021, Duesseldorf, Germany) and microinjected into the B6/FVB oocytes according to the standard procedures. Genotypes of the offspring were determined by PCR analysis using primers (5′-AAGGACGACGGCAACTACAAG-3′, and 5′-AAGGACGACGGC AACTACAAG-3′) to amplify a 378 bp GFP fragment.

### 2.2. Mouse Breeding and Tamoxifen Administering

All mice were maintained on an outbred CD1 genetic background and were housed in the animal facility at the Southeast University. All experimental procedures followed the guidelines approved by Southeast University. The day the vaginal plug was found at noon was considered E0.5. To overexpress Foxg1 in the hem, the *CAG-loxp-stop-loxp-Foxg1-IRES-EGFP* males were crossed with *Fzd10CreER^TM^* females and Tamoxifen (TM, Sigma, T5648, St. Louis, MO, USA) was administered by oral gavage to pregnant females at E10.5. TM was dissolved in corn oil (Sigma-Aldrich, C8267, St. Louis, MO, USA) at a concentration of 15 mg/mL and the dose administered was 75 μg/gm body weight.

### 2.3. Tissue Processing

E12.5 brains were rinsed with cold phosphate buffered saline (PBS) then fixed in 4% paraformaldehyde (PFA, Sigma-Aldrich, 441244, St. Louis, MO, USA) at 4 °C overnight, while E18.5 brains were perfused by 4% PFA and post-fixed in 4% PFA at 4 °C for 12–16 h. Brains were cryoprotected in 30% sucrose, embedded in OCT. The tissues applied in situ hybridization were rinsed with DEPC-PBS and the sucrose were dissolved in DEPC-PBS. The coronal sections (12 µm thick) were obtained using a Leica cryostat (CM 3050S) and stored at −70 °C until use.

### 2.4. In Situ Hybridization

Digoxigenin (DIG) UTP-labelled riboprobes were used. Probes were obtained by PCR amplification. The in situ hybridization was performed as previously described [19,20].

### 2.5. Immunofluorescence

Immunofluorescence was performed as previously described [21,22]. The primary antibodies and dilutions were as follows: anti-Calretinin (Millipore, AB5054, 1:500, Billerica, MA, USA); anti-Ctip2 (Abcam, ab18465, 1:2000, Cambridge, MA, USA); anti-Foxg1 (Abcam, ab18259, 1:1000, Cambridge, MA, USA); anti-GFP (Abcam, ab13970, 1:1000, Cambridge, MA, USA); anti-Lhx2 (Abcam, ab184337, 1:500, Cambridge, MA, USA); anti-P73 (Abcam, ab40658, 1:500, Cambridge, MA, USA); anti-Prox1 (Millipore, AB5475, 1:1000, Billerica, MA, USA); and anti-Reelin (Millipore, MAB5364, 1:1000, Billerica, MA, USA). The secondary antibodies used were Alexa Fluro 488 donkey anti-chicken (Jackson Lab, 703-545-155, 1:500, West Grove, PA, USA), Alexa Fluor 546 donkey anti-rabbit (Life, A10040, 1:500, Gaithersburg, MD, USA), Alexa Fluro 647 donkey anti-rabbit (Life, A31573, 1:500, Gaithersburg, MD, USA), Alexa Fluor 546 donkey anti-rat (Life, A10040, 1:500, Gaithersburg, MD, USA), CF 568 donkey anti-rat (Sigma-Aldrich, SAB4600077, 1:500, St. Louis, MO, USA), CF 633 donkey anti-rat (Sigma-Aldrich, SAB4600133, 1:500, St. Louis, MO, USA), and Alexa Fluro 647 donkey anti-mouse (Invitrogen, A21236, 1:500, Carlsbad, CA, USA).

## 3. Results

### 3.1. Forced Expression of Foxg1 in the Cortical Hem and Impaired Development of the DG

**Figure 1:**
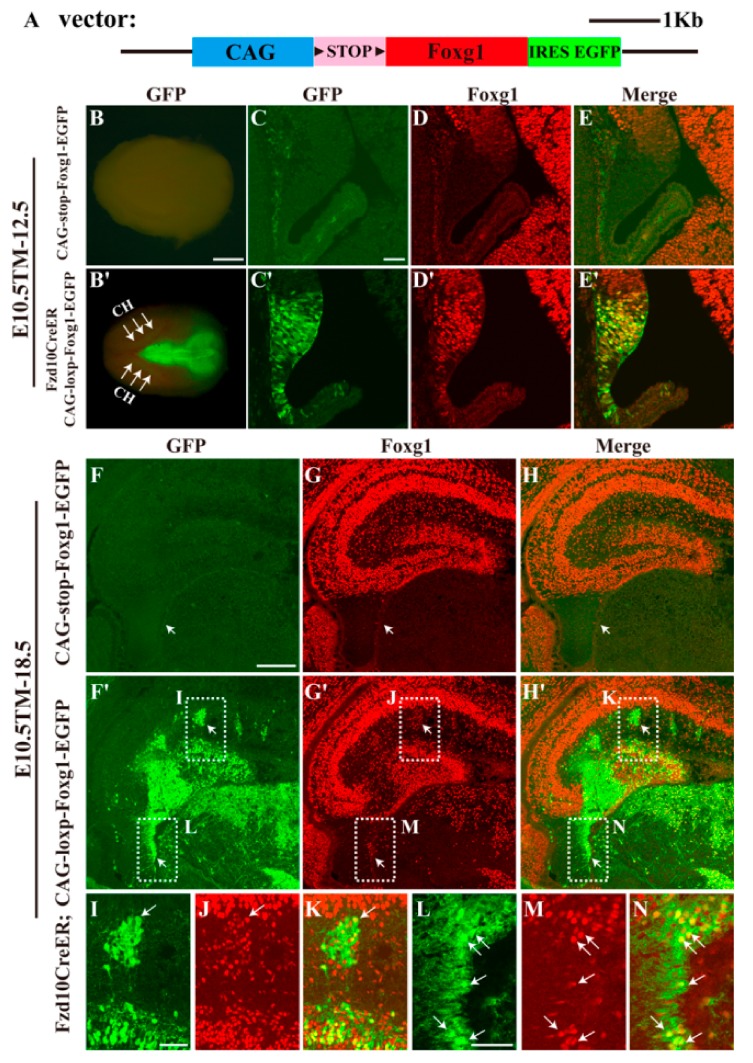
Forced expression of Foxg1 in the cortical hem and abnormal development of the dentate gyrus. (**A**) Schematic representation of the construction of the transgenic vector. (**B**,**B′**) Dorsal view of an E12.5 *Fzd10CreER^TM^;CAG-loxp-Foxg1-IRES-EGFP* hemisphere after E10.5 TM-induction. Reporter EGFP specifically labelled the hem and the dorsal thalamus where endogenous *Fzd10* is expressed. Arrows in B’ indicate the GFP-expressing cortical hem (CH). (**C**–**E′**) Double immunostaining for GFP with Foxg1 at E12.5 coronal sections. In controls (**C**–**E**), GFP was not expressed, and Foxg1 expression is excluded from the hem, no co-localization of Foxg1 and GFP to be detected. In (**C′**–**E**′), Foxg1 was ectopically expressed in the hem and co-expressing GFP. (**F**–**H**) At E18.5, in the *CAG-loxp-stop-loxp-Foxg1-IRES-EGFP* control, no GFP was detected, and Foxg1 was expressed in both CA and DG regions but specifically excluded from the fimbria. (**F′**–**H′**) In addition to the normal DG granule cells, the expression of Foxg1 was also detected in GFP^+^ cells. There were several clusters that were distributed outside the DG region. A mass of GFP^+^ tissue was ectopically located at the area between the fimbria and the developing DG. (**I**–**N**) Magnified views of boxed areas in (**F**′–**H′**) showing the ectopic expression of Foxg1 in clusters and the fimbria. Scale bars: (**B**,**B′**) 2 mm; (**C**–**H′**) 200 μm; (**I**–**N**) 50 μm.

To elucidate the role of Foxg1 in the maintaining of the hem from E10.5 onwards, we first generated a transgenic mouse line in which full-length *Foxg1* cDNA was driven by the *CAG* promoter. A Floxed stop codon and a reporter *IRES-EGFP* fragment were also introduced into the transgenic vector (Figure 1A). Previously, we have reported that one of the Wnts receptors, *Frizzled10* (*Fzd10*), is specifically expressed in the hem [23] and generated transgenic mouse lines of *Fzd10-TauLacZ* and *Fzd10-CreER^TM^* using 5′ untranslated regions of the *Fzd10* gene demonstrated that a large population of CR cells are derivatives of Fzd10-positive cells in the hem [23,24,25]. In this study, the overexpression of Foxg1 in the hem was achieved by crossing *Fzd10CreER^TM^* with the *CAG-loxp-stop-loxp-Foxg1-IRES-EGFP* line combined with tamoxifen induction. We first performed tamoxifen administration in *Fzd10CreER^TM^;CAG-loxp-stop-loxp-Foxg1-IRES-EGFP* at E10.5, the time window when the formation of the hem is almost completed. As shown in Figure 1B–C′, at E12.5, the reporter EGFP specifically labelled the hem and the dorsal thalamus where endogenous *Fzd10* is expressed. Strong ectopic expression of Foxg1 in the hem was observed to be completely co-localized with EGFP (Figure 1C′–E′), indicating a successful forced expression of Foxg1.

Since the hem is critical for the development of the hippocampus, we then analysed the hippocampal morphology at E18.5. In the *CAG-loxp-stop-loxp-Foxg1-IRES-EGFP* control brain, no GFP was detected (Figure 1F), and Foxg1 was expressed in both cornu ammonis (CA) and DG regions, but specifically excluded from the fimbria, which contains the residue of the hem (Figure 1G). However, in the transgenic *Fzd10CreER^TM^;CAG-loxp-Foxg1-IRES-EGFP* mice, in the future dentate blade, besides the granule cells, many GFP^+^ cells were detected to be Foxg1^+^. Several GFP^+^ clusters were found to be distributed outside the DG region (Figure 1F′–H′, I–J). Cells in clusters displayed strong ectopic expression of Foxg1 (Figure 1I–K). A mass of hem-derived GFP^+^ tissue was ectopically located at the area between the fimbria and the developing DG, and Foxg1 was detected to be located in cell bodies within the mass with the processes of these cells were Foxg1^−^ (Figure 1F′–H′). The fimbria, the structure mainly consisting of neuronal projections, was also found to contain Foxg1-expressing cells migrating towards the future DG (Figure 1L–N). Thus, Foxg1 was successfully overexpressed in the hem and its derivatives.

### 3.2. Most Hem-Derived Cells Lost CR Cell Fate and Mis-Distributed in the DG Area

**Figure 2:**
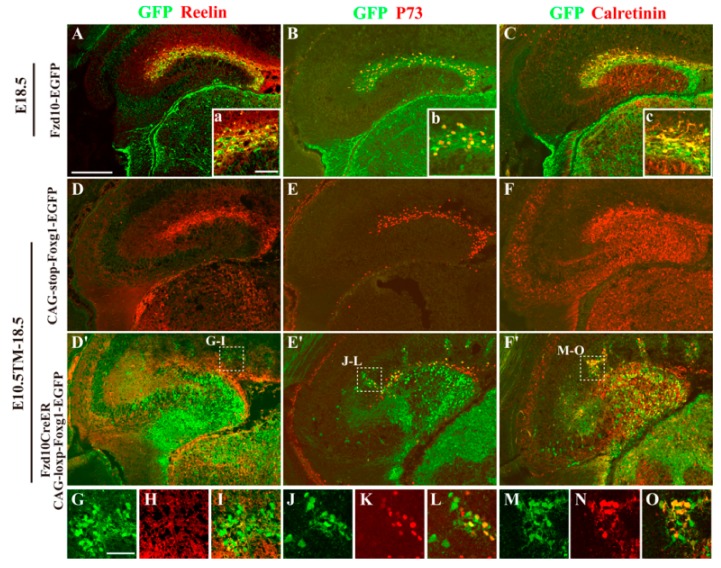
Most hem-derived cells lost CR cell fate and mis-distributed in the DG area. (**A**–**C**) Double immunofluorescence analysis of GFP and Reelin (**A**), P73 (**B**), and Calretinin (**C**) on coronal brain sections of *Fzd10-EGFP* brains at E18.5. The hem-derived GFP cells are both Reelin^+^, P73^+^ and CR^+^, indicating these are Cajal-Retzius cells. (**a**–**c**) Magnified views of the double immunostaining in the hippocampal MZ. (**D**–**F**) Confocal images of immunofluorescence of GFP and Reelin (**D**), P73 (**E**), and Calretinin (**F**) on *CAG-loxp-stop-loxp-Foxg1-IRES-EGFP* control brain when TM was administered at E10.5. (**D′**,**E′**) In the Foxg1 overexpressed transgenic brain, only a small portion of GFP^+^ cells distributed to the MZ and co-expressed Reelin and P73. The majority of GFP^+^ cells were Reelin^–^ and P73^–^ and dispersed within the DG area. (**F′**) In addition to immature granule neurons and CR cells, Calretinin was also found to be expressed in most GFP^+^ cells within clusters, as well as these dispersed cells in the DG area. (**G**–**O**) Magnified views of boxed areas in (**D′**–**F′**). Scale bars: (**A**–**F′**) 200 μm; (**G**–**O**) 50 μm.

By cell linage mapping, we previously demonstrated that a distinct population of CR cells originated from Fzd10-expressing progenitors in the hem and preferentially distributed to the hippocampal marginal zone (MZ) [23,24,26]. To examine whether the mis-located GFP^+^ cells in *Fzd10CreER^TM^;CAG-loxp-Foxg1-IRES-EGFP* still owned the CR cell fate, immunostaining of anti-Reelin and P73, two specific markers for the hem-derived CR cells, was then carried out. Since Fzd10 itself is only expressed in the hem but not expressed in its derivatives of CR cells, and previously we have shown the reporter gene *Tau-LacZ* driven by *Fzd10* promoter can act as a linage tracer during the embryonic developmental stages [25], the mouse lines of *Fzd10-EGFP* and *CAG-loxp-stop-loxp-Foxg1-IRES-EGFP* were used as controls in this study. In the *Fzd10-EGFP* control, GFP^+^ cells were observed to originate from the hem and populated only to the MZ, and these cells are both Reelin^+^ and P73^+^ (Figure 2A,B). In the *CAG-loxp-stop-loxp-Foxg1-IRES-EGFP* control, Reelin^+^ and P73^+^ CR cells are also observed to be distributed in the MZ and the migration route from the fimbria to the developing DG (Figure 2D,E). However, in the Foxg1 overexpressed transgenic brain, only a small portion of GFP^+^ cells distributed to the MZ and co-expressed Reelin and P73 (Figure 2D′,E′). The majority of GFP^+^ cells were Reelin^−^ and P73^−^ and dispersed within the DG area (Figure 2D′,E′), suggesting most of them lost CR cell fate. Interestingly, GFP^+^ clusters outside the DG showed heterogeneous property. Some cells within clusters expressing Reelin and P73, displaying a CR cell fate, while many cells were Reelin^–^ and P73^–^, indicating the clusters contained different cell types (Figure 2G–L).

To further demonstrate the cell fate alteration caused by Foxg1 forced expression, immunostaining of anti-Calretinin, a calcium binding protein commonly used as a marker for CR cells and immature granule neurons [27,28,29], was employed. As shown in Figure 2C,F,F′,M–O, in both controls, Calretinin was expressed in immature granule neurons in the developing DG and CR cells located in the MZ (Figure 2C,F), while, in the *Fzd10CreER^TM^;CAG-loxp-Foxg1-IRES-EGFP* brain, besides immature granule neurons and CR cells, Calretinin was also found to be expressed in most GFP^+^ cells within clusters as well as these dispersed cells in the DG area (Figure 2F′,M–O). These Reelin^−^P73^−^GFP^+^Calretinin^+^ cells might represent a cell population of immature granule neurons.

### 3.3. Hem-Derived CR Cells Switched Their Fates into Dentate Granule Neurons

**Figure 3:**
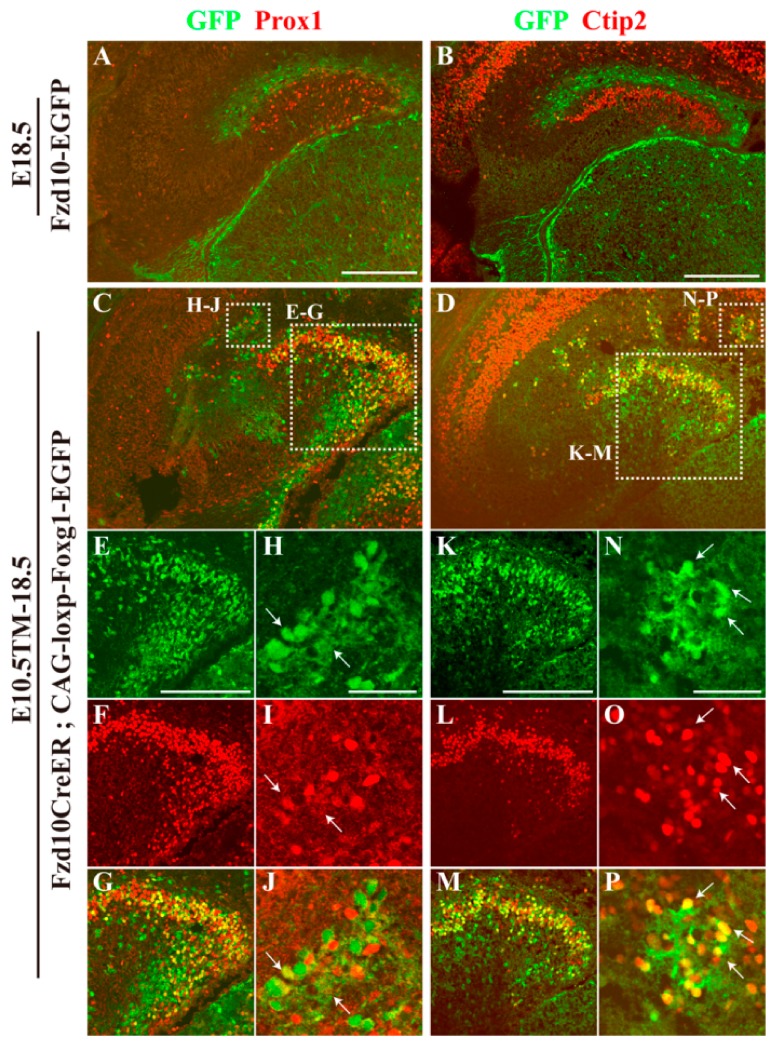
Hem-derived CR cells switched their fates into dentate granule neurons. (**A**,**B**) Double immunofluorescence analysis of GFP and Prox1 (**A**), Ctip2 (**B**) on coronal brain sections of *Fzd10-EGFP* embryos at E18.5. Hem-derived GFP cells are negative for Prox1 and Ctip2. (**C**,**D**) Confocal images of immunofluorescence of GFP and Prox1 (**C**), Ctip2 (**D**) on coronal E18.5 brain sections when TM was administered at E10.5. The ectopically-located hem-derived cells within the DG transformed to the granule neurons. (**E**–**P**) Magnified views of boxed areas in (**C**,**D**). Arrows in (**I**,**J**) indicate the GFP^+^; Prox1^+^ cells, arrows in (**O**,**P**) indicate the GFP^+^;Ctip2^+^ cells. Scale bars: (**A**–**D**) 200 μm; (**E**–**P**) 50 μm.

To further analyse the feature of the population of Reelin^−^P73^−^GFP^+^Calretinin^+^ cells, immunostaining of anti-Prox1 and Ctip2, two transcription factors that are critical for the development of granule neurons [30,31], was carried out. At E18.5, in the *Fzd10-EGFP* control, there was no co-localization of GFP with Prox1 or Ctip2, and Prox1^+^ and Ctip2^+^ granule neurons were located inside the DG MZ, forming the upper blade of the DG (Figure 3A,B). On the contrary, in the *Fzd10CreER^TM^;CAG-loxp-Foxg1-IRES-EGFP* brain, both Prox1 and Ctip2 were found to be expressed in clusters outside the DG (Figure 3C,D,H–J,N–P). Within the DG, many GFP^+^ cells co-expressed Prox1 and Ctip2 (Figure 3C–G,K–M), demonstrating these ectopically-distributed hem-derived cells transformed into granule neurons.

**Figure 4:**
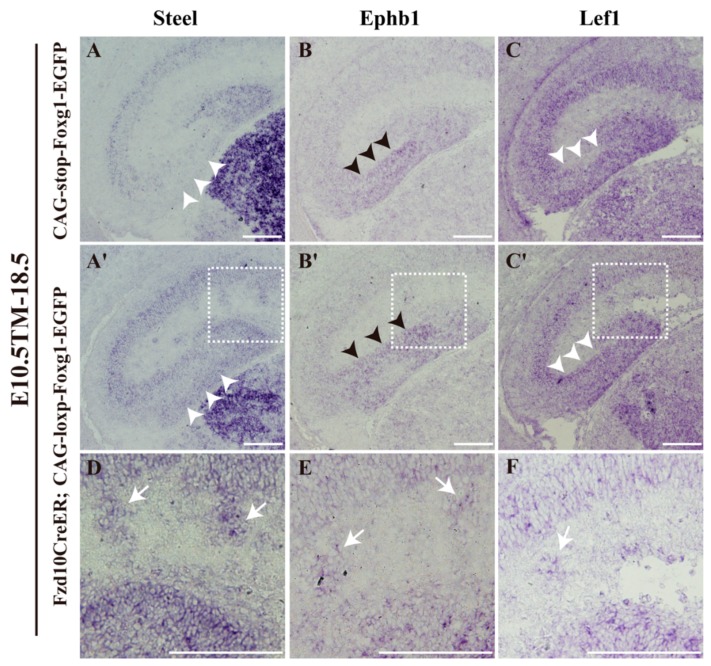
Misdistributed hem-derived cells outside the DG has the property of DG granule neurons. (**A**–**C′**) In situ hybridization assay of *Steel* (**A**,**A′**), *Ephb1* (**B**,**B′**), and *Lef1* (**C**,**C′**) on coronal sections of E18.5 *CAG-loxp-stop-loxp-Foxg1-IRES-EGFP* (**A**–**C**) and *Fzd10CreER^TM^;CAG-loxp-Foxg1-IRES-EGFP* embryos (**A′**–**C′**) showing increased staining for *Steel* ((**A′**), white arrowheads) and a little thicker staining for *Ephb1* ((**B′**), black arrowheads) on the migration route. Thicker staining for *Lef1* was also observed in the migration route ((**C′**), white arrowheads). (**D**–**F**) Magnified views of boxed areas in (**A′**–**C′**). Short white arrows indicate the ectopically located clusters. Scale bars: 200 μm.

Steel and Ephb1 have been used to label developing granule cells in the DG [32,33,34]. To confirm the cell fate transformation, in situ hybridization of *Steel* and *Ephb1* was performed on E18.5 brains after tamoxifen administration at E10.5. As shown in Figure 4A,B, in the control, strong staining for *Steel* was detected in the DG, and *Ephb1* was strongly detected in migrating granule cells. In Foxg1 overexpressed transgenic brains, in the migration route increased staining of *Steel* was observed (Figure 4A,A′), and the staining of *Ephb1* seemed a little thicker than that of the controls. Ectopic staining of *Steel* and *Ephb1* was also detected in clusters outside the DG (Figure 4A′,B′,D,E). Since *Lef1*, a mediator in the Wnt signalling pathway, plays an important role during the development of the DG [7], we then examined its expression. Compared to the control, in *Fzd10CreER^TM^;CAG-loxp-Foxg1-IRES-EGFP* brain, similar to that of *Steel* and *Ephb1*, clusters also displayed ectopic strong staining for *Lef1* (Figure 4C–C′,F). A little thicker staining of *Lef1* in the migration route was also detected (Figure 4C,C′). Taken together, our data demonstrated hem-derived CR cells switched their fates into dentate granule neurons.

### 3.4. Normal Morphology of the Hem and Ectopic Expression of Lhx2 after Foxg1 Overexpression

**Figure 5:**
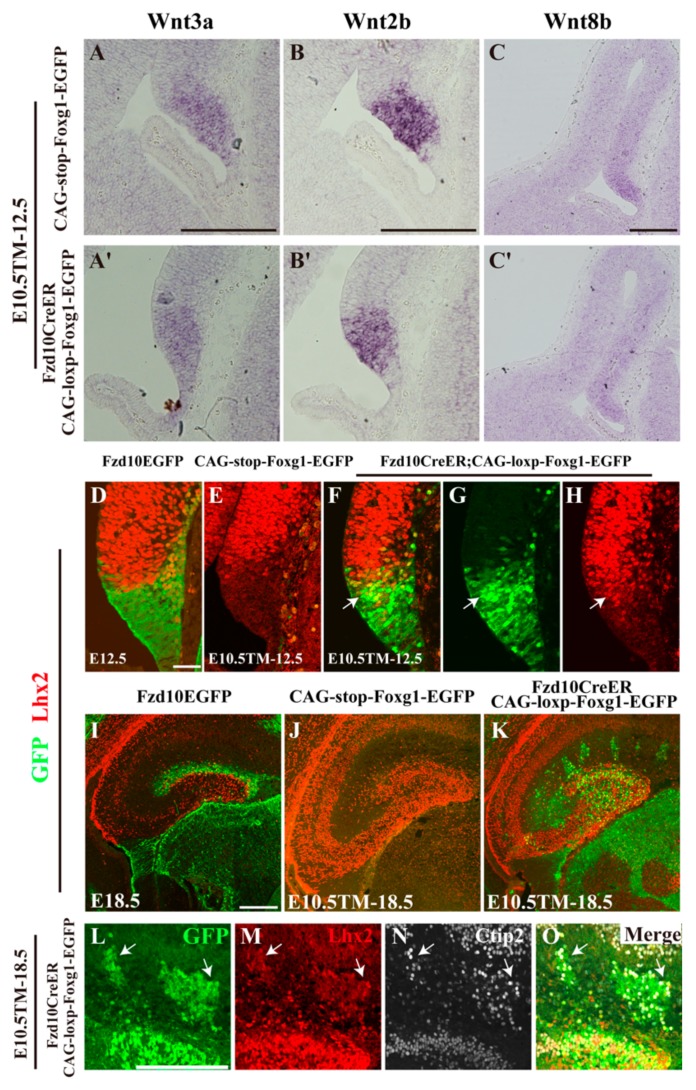
Normal morphology of the hem and ectopic expression of Lhx2. (**A**–**C′**) In situ hybridization assay of *Wnt3a* (**A**,**A′**), *Wnt2b* (**B**,**B′**), and *Wnt8b* (**C**,**C′**) on coronal sections of E12.5 *CAG-loxp-stop-loxp-Foxg1-IRES-EGFP* (**A**–**C**) and *Fzd10CreER^TM^; CAG-loxp-Foxg1-IRES-EGFP* embryos (**A′**–**C′**). (**D**–**K**) Double-immunofluorescence analysis of GFP with Lhx2 at E12.5 (**D**–**H**) and E18.5 (**I**–**K**). (**L**–**O**) Triple immunostaining for GFP/Lhx2/Ctip2 in E12.5 *Fzd10CreER^TM^; CAG-loxp-Foxg1-IRES-EGFP* brains. The arrows show the triple-labelled cells. Scale bars: 200 μm ((**D**–**G**) 50 μm).

Next, we analysed whether the hem itself was affected by Foxg1 overexpression. First, in situ hybridization for *Wnt3a* and *Wnt2b*, two members of the Wnt family, which are specifically expressed in the hem, was performed. Interestingly, the morphology of the hem delineated as the staining of *Wnt3a* and *Wnt2b* seemed normal, while the expression level of *Wnt3a* and *Wnt2b* were slightly reduced (Figure 5A–B′). We then examined *Wnt8b*, another Wnt family member normally expressed in the medial pallium, and found *Wnt8b* was also downregulated (Figure 5C,C′), consistent with previously-reported results that *Foxg1* suppresses *Wnt8b* [35].

Previous studies have shown that Lhx2-expressing tissues near the hem can be specified into the hippocampi [8]. Considering the transformation of CR cells into dentate granule neurons in our transgenic mice, we suspected that the expression of Lhx2 was induced upon forced expression of Foxg1 in the hem. To confirm the hypothesis, double immunostaining of anti-Lhx2 with GFP was carried out. At E12.5, in the *Fzd10-EGFP* control, Lhx2 was strongly expressed in the medial pallium, but specifically excluded from the GFP^+^ hem area, there was no co-localization of Lhx2 with GFP (Figure 5D). The same result was obtained in the *CAG-loxp-stop-loxp-Foxg1-IRES-EGFP* control. There was no Lhx2 expression detected in the hem either (Figure 5E). However, in the *Fzd10CreER^TM^;CAG-loxp-Foxg1-IRES-EGFP* transgenic line, Lhx2 was found to be ectopically expressed in the GFP^+^ hem (Figure 5F–H), demonstrating that Foxg1 is sufficient to induce the expression of Lhx2. Interestingly, it seemed that the expression of Lhx2 was only induced in the dorsal Wnt-rich part of the hem, while, in the ventral BMP-rich subarea, Lhx2 expression was not detected (Figure 5F–H), although Foxg1 was ectopically expressed there as well (Figure 1D′,E′). At E18.5, compared with Lhx2 expression observed in the DG area in both *Fzd10-EGFP* and *CAG-loxp-stop-loxp-Foxg1-IRES-EGFP* controls (Figure 5I,J), in the Foxg1 overexpressed transgenic brain, ectopic Lhx2 was additionally observed to be expressed in the clusters outside the DG, and co-localized with GFP and Ctip2 (Figure 5K–O). Taken together, by inducing the expression of Lhx2, overexpressed Foxg1 transformed the hem-derived CR cells into granule neurons.

## 4. Discussion

The cortical hem is one of the cortical organizers, which plays critical roles in the pattern formation of the telencephalon, and it is particularly required for the induction of the hippocampus [5,6,8]. Loss of the hem severely impairs the dorsomedial neocortex [5]. The hem is also one of the sources of CR cells [9,36]. A subpopulation of CR cells originates from the hem and settle in the media-caudal surface of the developing telencephalon [24,26]. However, how the hem is regulated remains unclear. To gain insight of the mechanism underlying the maintenance of the hem, in this study, Foxg1 was ectopically expressed in the hem from E10.5 onwards. Hem-derived CR cells switched their fate into dentate granule neurons after forced expression of Foxg1. However, the morphology of the hem seemed normal and only showed slightly down-regulated Wnts. We also found Lhx2 expression was induced, which may be responsible for the cell fate transformation. Our result will help to understand the mechanism of the maintenance of the hem.

Foxg1 has been shown to play important roles in pattern formation, cell proliferation and cell specification [15,22,37,38,39]. Here, by overexpression of Foxg1 in the hem from E10.5 onwards, we detected a cell fate switch of the hem-derived CR cells to dentate granule neurons, accompanied with ectopic expression of Lhx2. Previous study has reported that loss of Foxg1 leads to a reduction of Lhx2 expression, and *Foxg1* can directly bind to *Lhx2* locus [16]. In this study, we demonstrated that forced expression of Foxg1 is sufficient to induce Lhx2 expression in the hem. It has been reported that Lhx2-expressing cells can be specified into hippocampal cells and this only happens in tissues adjacent to the hem [16]. Here, induced expression of Lhx2 in our transgenic mice may be responsible for the transformation of CR cells to their granular fate. Interestingly, we have observed that the induction of Lhx2 occurs only in the dorsal part of the hem. Although Foxg1 is also overexpressed in the ventral part, the expression of Lhx2 is not detected. Since the dorsal part of the hem is Wnt rich, while the ventral is BMP rich, one possible explanation is that other signalling pathways are also involved in the maintenance of the hem. Further studies are required to elucidate the mechanism underlying the hem heterogeneity.

Early constitutive loss of Foxg1 (around E8.5–9.0) results in severe expansion of the hem, while removal of Foxg1 at E13.5 has no obvious influences [13,16], suggesting that Foxg1 may regulate the hem in a spatiotemporal manner. In this study, Foxg1 was forced to be expressed in the hem from E10.5 onwards. To our surprise, the morphology of the hem seemed normal, and *Wnt3a* and *Wnt2b* were only slightly down-regulated, indicating the suppression of *Foxg1* on the hem and Wnt signalling is gradually weakened. *Wnt8b*, expressed in the medial pallium, was down-regulated, consistent with previously-reported results [35]. We also found *Lef1*, the downstream transcription factor in the Wnt signalling pathway, was ectopically expressed in clusters outside the DG, suggesting that Wnt signalling may contribute to the cell fate switch as well.
